# “Do not look at me like that”: Is the facial expression score reliable and accurate to evaluate pain in large domestic animals? A systematic review

**DOI:** 10.3389/fvets.2022.1002681

**Published:** 2022-12-06

**Authors:** Carola Fischer-Tenhagen, Jennifer Meier, Alina Pohl

**Affiliations:** ^1^German Centre for the Protection of Laboratory Animals (Bf3R), German Federal Institute for Risk Assessment (BfR), Berlin, Germany; ^2^Clinic of Animal Reproduction, Freie Universität Berlin, Berlin, Germany

**Keywords:** grimace scales, assessment, pain, facial action, large animal

## Abstract

**Introduction:**

Facial expression scoring has proven to be useful for pain evaluation in humans. In the last decade, equivalent scales have been developed for various animal species, including large domestic animals. The research question of this systematic review was as follows: is facial expression scoring (intervention) a valid method to evaluate pain (the outcome) in large domestic animals (population)?

**Method:**

We searched two databases for relevant articles using the search string: “grimace scale” OR “facial expression” AND animal OR “farm animal” NOT “mouse” NOT “rat” NOT “laboratory animal.” The risk of bias was estimated by adapting the Quality Assessment of Diagnostic Accuracy Studies (QUADAS) checklist.

**Results:**

The search strategy extracted 30 articles, with the major share on equids and a considerable number on cows, pigs, and sheep. Most studies evaluated facial action units (FAUs), including the eye region, the orbital region, the cheek or the chewing muscles, the lips, the mouth, and the position of the ears. Interobserver reliability was tested in 21 studies. Overall FAU reliability was substantial, but there were differences for individual FAUs. The position of the ear had almost perfect interobserver reliability (interclass coefficient (ICC): 0.73–0.97). Validity was tested in five studies with the reported accuracy values ranging from 68.2 to 80.0%.

**Discussion:**

This systematic review revealed that facial expression scores provide an easy method for learning and reliable test results to identify whether an animal is in pain or distress. Many studies lack a reference standard and a true control group. Further research is warranted to evaluate the test accuracy of facial expression scoring as a live pen side test.

## Introduction

Reliable and accurate pain assessment is necessary for pain management and, specifically, the impact of interventions on animals in experiments. Only if pain is correctly recognized and classified, it can be successfully managed. Pain is defined as “an unpleasant sensory and emotional experience associated with, or resembling that associated with, actual or potential tissue damage” (1, 2). Pain not only is a question of the severity of trauma or tissue damage but also has a time dimension. Acute pain occurs in injuries or specific diseases and is associated with the activation of the sympathetic nervous system. Chronic pain persists for more than 3 months and is considered a disease state (3). In addition, pain also has an emotional and individual component. Therefore, pain is a subjective experience with multiple dimensions, all of which can have an influence on individual pain perception and expression. To estimate the pain sensation of the human individual patient, a numerical or visual rating scale from 1 to 10 was introduced to improve adequate pain management (4). Animals cannot verbally communicate their pain experience. Therefore, the gold standard for measuring pain in humans is not available in animals.

Current methods for assessing pain in animals focus on changes in behavior and physiology. Animals in pain feed less, play less, and have a change in activity and lying behavior (5, 6). The release of glucocorticoids (7), the change in heart rate variability (8), or the variation in the composition of immune cells (9) are useful physiological parameters for assessing aversive situations. However, on-farm or pen side pain identification techniques should rely on immediate rather than retrospective indicators of pain. This ensures that humane intervention can be applied promptly without leaving animals in distress for an extended period of time (10).

In non-verbal humans, like infants, facial expressions provide a reliable indicator of pain (11, 12). Facial expression is the measure of changes in the face or in groups of muscles, known as “action units” in relation to a stimulus. Ekman (13) developed the Facial Action Coding System (FACS). This system enabled trained persons to code over 40 distinct muscle movements in the face (14). The benefits of externalizing pain through facial expressions are thought to be evolutionary and effective in increasing the chances of survival by inducing empathy in other individuals (15, 16).

Facial expressions have been shown to be consistent during the induction of pain by various modalities of nociceptive stimulation in humans. The human pain face comprises five action units: brow lowering, lid tightening, wrinkled nose, raised upper lip, and eye closure (17). Darwin (18) also observed that animals express emotions through facial expressions similar to humans. Across the different species, there are similar facial movements and action units expressed in the presence of pain (19). Thus, facial expressions are considered honest signals of the affective state and pain intensity (20).

In 2010, Langford et al. (21) introduced a facial expression score to assess pain in mice by comparing the facial expressions of painless and painful animals. Since then, similar comparable “grimace scales” or “facial expression scores” were developed and reported for various species, such as rats (22), rabbits (23), ferrets (24), sheep (25), horses (26), pigs (27), cattle (28), and cats (29). In most of these studies, scientists produced frames out of videos pre and post painful experiences in animals. Scientists could demonstrate that observers blinded to treatment could identify specific pain faces and scored frames of animals with pain higher than animals without pain.

Cows and sheep are often described as especially stoic and showing no pain (30). Modern cows, extensively managed ruminants, and their wild ancestors are still considered prey species. It is thought that showing evidence of injury could attract potential predators. As they do not inherently portray pain, it makes it even more difficult for humans to determine their welfare needs. Therefore, pain assessment in farm animals is especially critical. Several studies report evidence that facial expressions are valid and reliable for evaluating pain in farm or large domestic animals (25, 26).

The objectives of this systematic review were to summarize and categorize the results of recent papers on the facial expression score in large domestic animals. Our specific research question was: Is the facial expression score (intervention) a valid method to evaluate pain (the outcome) in large domestic animals (population)? We wanted to evaluate the risk of bias in these studies and compare the results in terms of reliability and accuracy. As a result, we wanted to identify the best practice for the use of facial expression scoring in large domestic animals, point out the flaws and challenges with this technique, and identify the need for further research in this field.

## Materials and methods

**Figure 1:**
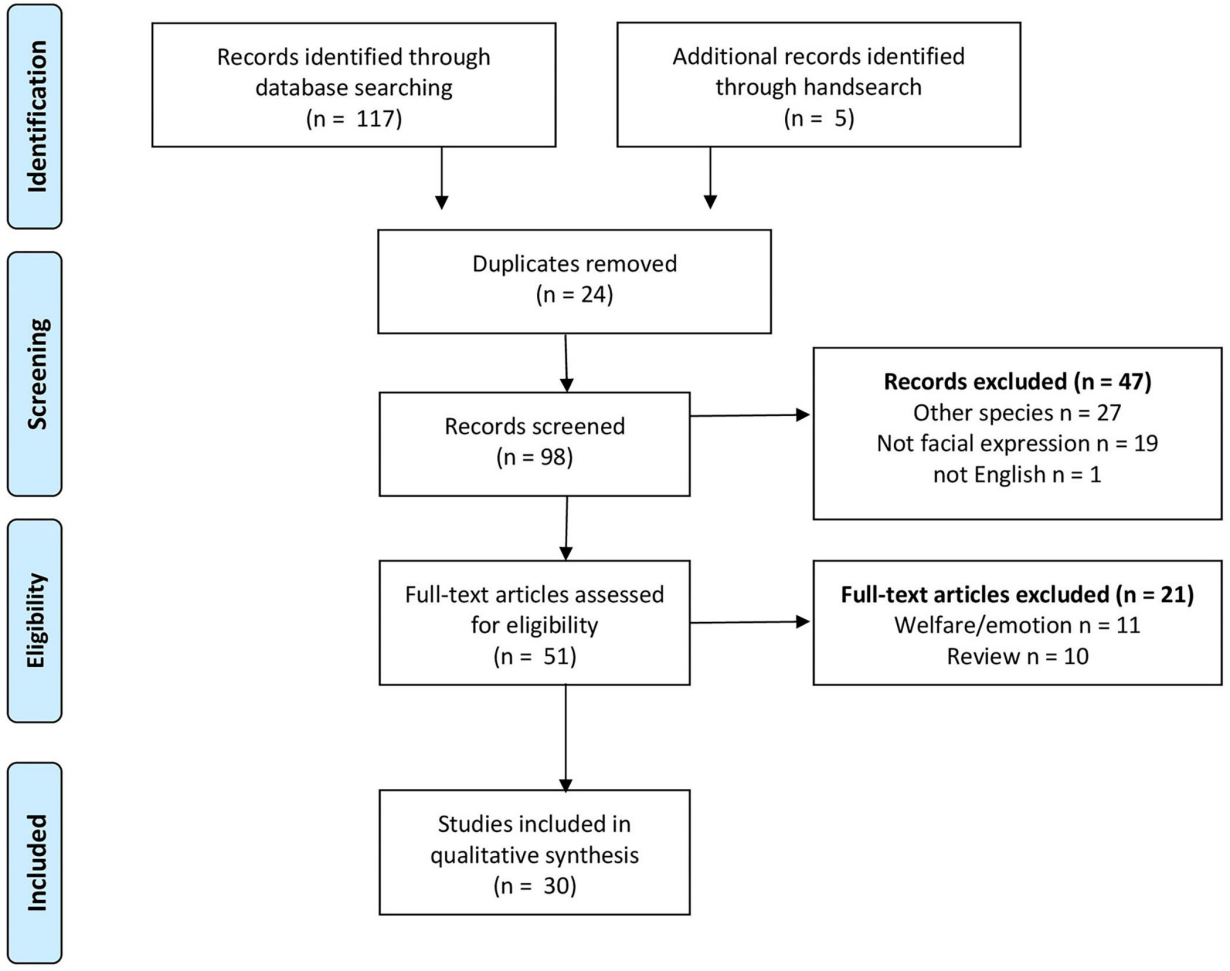
Preferred Reporting Items for Systematic reviews and Meta-Analyses (PRISMA) flow diagram with the selection process of relevant articles (31).

To identify the literature relevant to the question, we developed a search strategy in the PubMed (https://pubmed.ncbi.nlm.nih.gov) and Web of Science (https://apps.webofknowledge.com) databases including the following keywords: “grimace scale” OR “facial expression” AND “pain” AND “animal” OR “farm animal” NOT “mouse” NOT “rat” NOT “laboratory animal.” We searched the database on 20 January 2022. Relevant articles found in the reference list of retained articles were included as “hand search.” The selection strategy is illustrated in Figure 1.

The data collection process was performed by the first author (CFT) and crosschecked by JM and AP to ensure the integrity of the contents. Articles were screened by title and abstract, and we included articles with the full text published in peer-reviewed journals, written in English, and evaluating pain assessment with facial expressions in large domestic animals. We excluded studies evaluating emotions or welfare *via* facial expressions. Conference abstracts and articles with only an abstract in English were also excluded.

To categorize and evaluate the articles, we assessed them according to the following criteria: the type of study (clinical study, case report, and data-based study), species involved in the study, sample size, qualification and number of observers, reference (gold) standard, the method of observation (real time, video, and pictures), interobserver reliability test, additional pain assessment methods (composition pain score, behavioral assessment), the number of facial action unit (FAU) scored, and scale range. The reference standard in this review is defined as the best available method to establish the presence or absence of the target condition; a gold standard would be an error-free reference standard (32). Data were extracted into Microsoft Excel (version 2013; Microsoft Corp., Redmond, WA, USA). Descriptive and explorative statistics were performed using SPSS for Windows (version 22.0; SPSS, Inc.). For other methods of addressing study quality, such as sensitivity analysis, subgroup analysis, or meta-regression analysis, the included studies were too low in number and too diverse in design for meaningful investigations.

We assessed the risk of bias in the individual study by adapting the Quality Assessment of Diagnostic Accuracy Studies (QUADAS) checklist (33). The final checklist consists of 12 questions that evaluate items with a potential risk of bias (Figure 3). The percentage of studies with a low, high, or unclear risk of bias for the respective item was summarized in a bar chart. Initially, the assessment was independently done by CFT and AP. In case of disagreement (5% of the answers on the checklist), both authors found a consensus after reviewing the manuscripts again.

## Results

Our search strategy resulted in 117 articles from the databases. Five additional articles were retrieved by scanning the reference list of relevant articles. We excluded duplicates (*n* = 24), non-English articles (*n* = 1), reviews (*n* = 10), articles evaluating welfare or emotions (*n* = 11), articles not focusing on large domestic animals (*n* = 27), or articles where the grimace scale or facial expressions were not the objectives of the study (*n* = 19) (Figure 1). We included 30 articles for further evaluation. The general characteristics of the studies are summarized in Table 1.

**Table 1:** General characteristics of articles (*n* = 30) included in this review.

**First Author**	**Year**	**Species**	**number of animals**	**Type of study**	**Pain typ**	**Score Typ**	**Number of score criteria**	**number of observer**	**observation**
Coneglien	2020	Horse	33	Clinical study	Dental treatment	HGS	6	8	Real time
Dai	2020	Horse	N.A.	Proof of concept	N.A	HGS	6	206	Photo
Dalla Costa	2014	Horse	46	Clinical study	Castration	HGS	6	6	Frame from video
Dalla Costa	2016	Horse	10	Clinical study	Acute lamintis	HGS	6	6	Video and photo
Dalla Costa	2021	Horse	11	Clinical study	Castration	HGS	6	4	Frame from video
Diego	2016	Horse	21	Clinical study	Follicular puncture	HGS	3	N.A.	Real time
Dierendonck	2020	Donkey	254	Clinical study	Painful diseases	FAP	12	6	Real time
Dyson	2017	Horse	101	Observation	Lameness	FEEP	14	1	Photosgraph
Giminiani	2016	Pig	23	Clinical study	Tail docking, castration	PGS	10	30	Frame from video
Gleerup	2015	Bovine	139	Clinical study	Clinical disease	FEE	6	4	Real time
Gleerup	2015	Horse	6	Clinical study	Capiscain, tournique	Painface	6	1	Photo
Guesgen	2016	Sheep	18	Clinical study	Tail docking	Ear	4	5	Frame from video
Häger	2017	Sheep	14	Clinical study	Tibiatomy	SGS	3	6	Frame from video
Lencioni	2021	Horse	7	Observation	Castration	HGS		1	Photo
McLennan	2016	Sheep	113	Clinical study	Disease	SGS	6	6	Photo
Mullard	2017	Horse	30	Observation	Lameness	FEE	14	13	Photo
Muller	2019	Bovine	35	Clinical study	Hot iron branding	FEE	15	1	Frame from video
Navarro	2020	Pig	21	Clinical study	Farrowing	PGS	5	8	Frame from video
Orth	2020	Donkey	9	Clinical study	Castration	DGS	9	12	Photo
Rashid	2020	Horse	27	Observation	Disease	FACS	27	1	Video
VanLoon	2021	Horse	53	Clinical study	None	FAP	9	2	Real time
VanLoon	2021	Donkey	77	Clinical study	None	FAP	12	2	Real time
VanLoon	2019	Horse	77	Clinical study	Trauma, surgery	FAP	9	2	Real time
VanLoon	2015	Horse	50	Clinical study	Colic	FAP	9	4	Real time
Viscardi	2017	Pig	19	Clinical study	Tail docking, castration	GS	3	2	Frame from video
Viscardi	2021	Sheep	30	Clinical study	Laparatomy	GS	6	3	Photo
Viscardi	2019	Pig	120	Clinical study	Castration	GS	3	8	Photo
Viscardi	2018	Pig	60	Clinical study	Castration	GS	3	4	Photo
Vullo	2020	Pig	10	Clinical study	Castration	GS	3	3	Frame from video
Yamada	2021	Bovine	45	Clinical study	Dental treatment	FAU	4	nk	Photo

NA, not applicable; HGS, horse grimace scale; PGS, pig grimace scale; DGS, donkey grimace scale; SGS, sheep grimace scale; GS, grimace scale; FAP, facial assessment of pain; FEE:,facial expression ethogram; FACS, facial action coding system.

Twenty-eight studies included animals for data collection. The number of animals included ranged from 6 to 254, with a median of 30 and an interquartile range (IQR) of 43. Two-thirds of studies included animals undergoing general veterinary treatment; all other studies used animals explicitly for their experiment (experimental animals). Two utilized pictures/videos from previous studies for analysis. Species involved were horses (*n* = 14), pigs (sows *n* = 1 and piglets *n* = 5), sheep (adult *n* = 2 and lambs *n* = 2), cattle (*n* = 3), and donkeys (*n* = 3).

Most studies were designed as a clinical study (*n* = 27). Two studies performed specific data analysis, and one manuscript described a training program for learning facial expressions. Clinical studies were categorized as observational studies (*n* = 7), randomized clinical controlled studies (*n* = 5), case-control studies (*n* =12), and cohort studies (*n* = 3).

The number of observers included in this study was reported in 28 studies, ranging from 1 to 206 with a median of 4.0 and an IQR of 6. Observers in these studies were veterinarians or students of veterinary medicine (*n* = 13), animal scientists or animal professionals (*n* = 8), lay people (*n* = 1), or non-specified (*n* = 8).

The observation modes were real-time (*n* = 8), videos (*n* = 1), and photographs (*n* = 2). Two studies evaluated videos and photos, and 10 studies picked frames out of videos to score FAUs. The number of FAU scored ranged from 3 to 27 with a median of 6. The scale ranged from 2 (yes/no) to 4, including the options “don't know” or “cannot see.”

**Figure 2:**
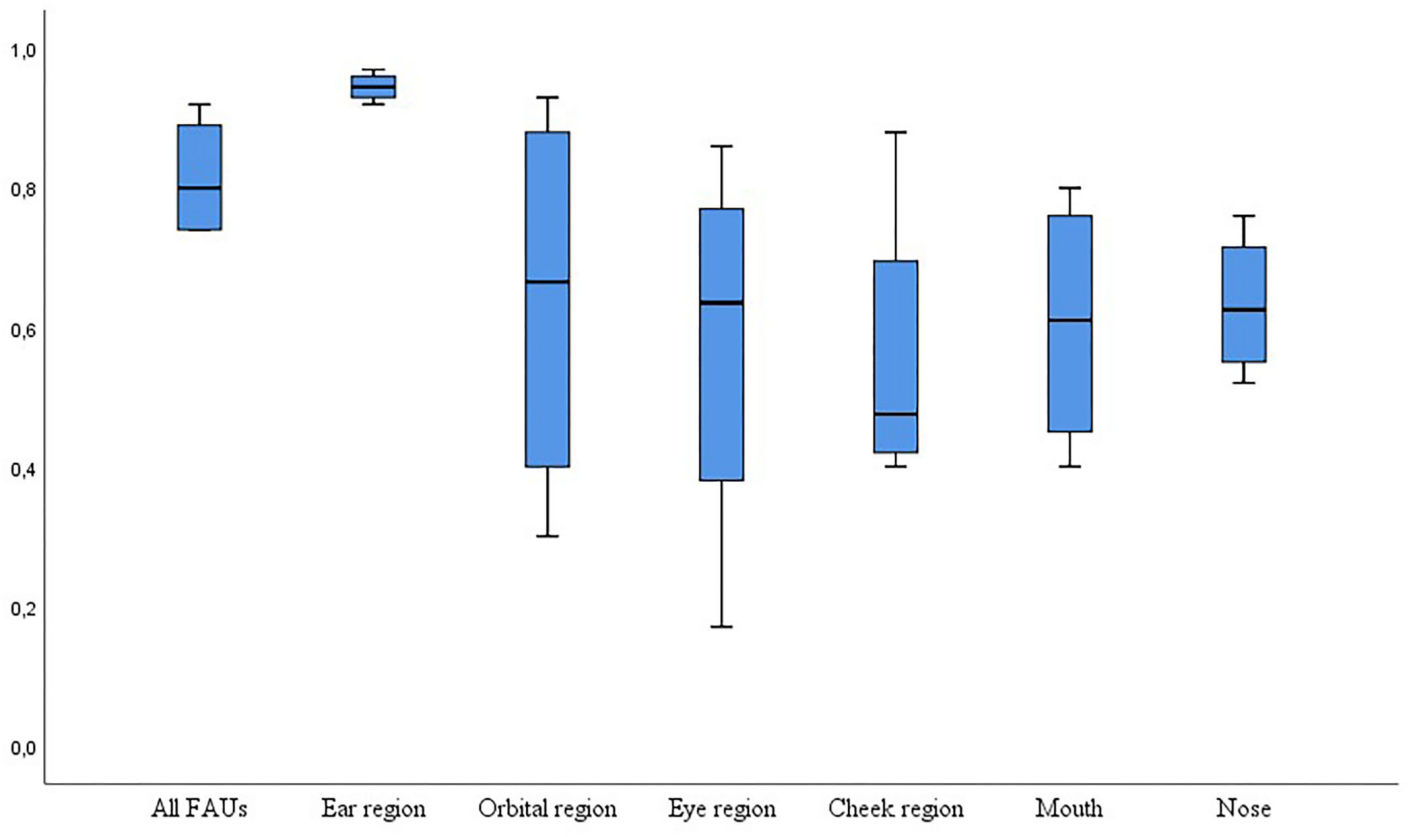
Box-plot graph for interclass coefficient (ICC) for interobserver reliability tested with Cohen's kappa coefficient (*y*-axis) for all facial action units scored (*n* = 19 studies) and for scores based on a single facial action unit (*n* = 11 studies). Agreement was “very good” with an ICC of 0.81–1.0, “good” with an ICC of 0.61–0.80, and moderate with an ICC of 0.41–0.6 (34).

Seventeen studies used or evaluated a grimace scale, whereas the rest of the studies evaluated pain by developing a facial expression ethogram with 1 to 27 FAUs. Twenty-one studies assessed and reported interobserver reliability for the scale including all FAUs. Interclass correlation (ICC, *n* = 19) and Kappa coefficient, Kendall, Cronbach's alpha (one each) were used as statistical methods (Table 2). The reported reliability coefficient ranged from 0.45 to 0.92. Eleven groups evaluated reliability for individual FAU ranging from 0.2 to 1.0 (Figure 2). Twenty-one studies evaluated differences in the grimace scale between animals in the pain and painless control groups, and 17 of these studies reported a significantly higher score for animals in pain. Three studies reported the accuracy of this method to identify pain ranging from 68.2 to 80%, and two groups reported the sensitivity and specificity of this method with 57/87.5% and 90.5/88%, respectively.

**Table 2:** Effect of treatment on facial expressions and statistical methods used for analysis.

**First Author**	**Year**	**Treatment**	**Effect**	**Statistics**
Coneglien	2020	Dental treatment	Lower pain score	Wilcoxon test
Dai	2020	N.A	N.A.	N.A.
Dalla Costa	2014	Castration	Effect on pain scores	GLENMIX; ANOVA
Dalla Costa	2016	Acute lamintis	Lower pain score	Wilcoxon signed rank test
Dalla Costa	2021	Castration	Higher pain score	Friedmantest; *post hoc* Bonferoni
Diego	2016	Follicular puncture	No effect	Mann–Whitney U
Dierendonck	2020	Painful diseases	Higher pain score	Mann–Whitney U
Dyson	2017	Lameness	Higher pain score	Mann–Whitney U
Giminiani	2016	Tail docking, castration	Difference only orbital tightening	Wilcoxon matched pair test
Gleerup	2015	Clinical disease	Higher pain scores	One-tailed *t*-test with Welch correction
Gleerup	2015	Capiscain, tournique	More pain face features	Wilcoxon signed rank test
Guesgen	2016	Tail docking,	Higher pain score	GLENMIX
Häger	2017	Tibiatomy	Higher pain score	ANOVA
Lencioni	2021	Castration	N.A.	N.A.
McLennan	2016	Disease	Higher pain score	Spearman's rank correlations
Mullard	2017	Lameness	N.A.	N.A.
Muller	2019	Hot iron branding	4 FAU with association to pain	McNemar test
Navarro	2020	Farrowing	N.A.	N.A.
Orth	2020	Castration	N.A.	N.A.
Rashid	2020	Disease	Chewing indicative for pain	paired *t*-test
VanLoon	2021	Chronic pain	Higher pain score only 1 day	Mann–Whitney U
VanLoon	2021	Chronic pain	Higher pain score	Mann–Whitney U
VanLoon	2019	Trauma, surgery	Higher pain score	Mann–Whitney U
VanLoon	2015	Colic	Higher pain score	Mann–Whitney U
Viscardi	2017	Tail docking, castration	Higher pain score	ANOVA
Viscardi	2021	Laparatomy	No effect on pain score	GLENMIX
Viscardi	2019	Castration	Effect on pain score	GLENMIX
Viscardi	2018	Castration	No effect on pain score	GLENMIX
Vullo,	2020	Castration	Higher pain score 6h post treatment	Paired Sample *t*-test
Yamada	2021	Dental treatment	Positive correlation for eye and above eye	Logistic regression, *post hoc* Tukey's test

NA, not applicable; ANOVA, analysis of variance; GLENMIX, general linear mixed model; FAU, facial action unit.

Four studies reported the values of facial expression scores pre- and post-intervention. Intervention is meant as the measure taken to provoke pain in the experiment. The horse grimace scale (maximum score 12) had a 3.5- and 2.3-point higher score after castration. Pigs (maximum score of 5) had a 1.14-point higher score after castration, and the sheep grimace scale (maximum score of 7) rose by 1.3 points after an orthopedic intervention. Different score systems, species, and the type of intervention did not allow any analysis of the effect of the intervention on the pain score.

**Figure 3:**
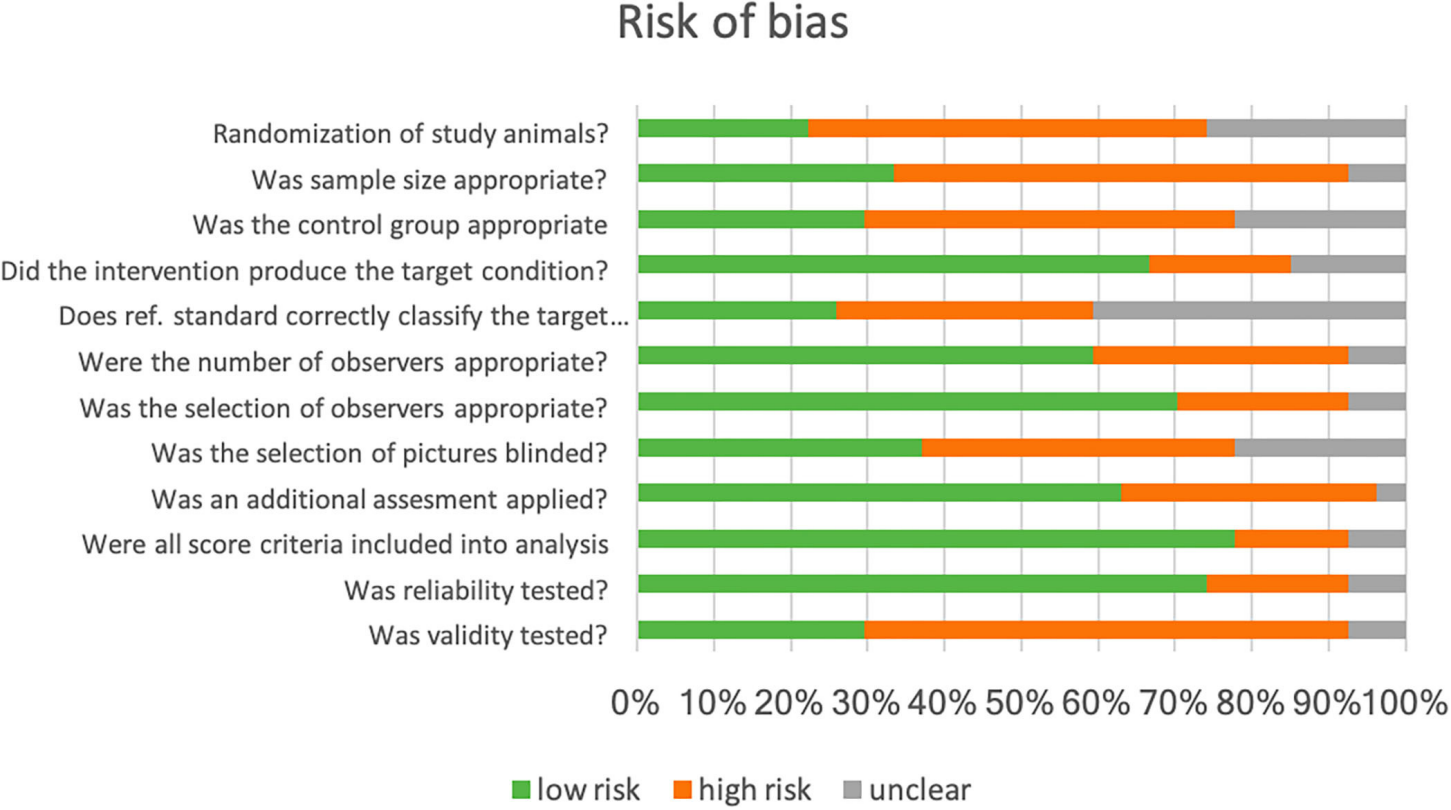
Bar chart for the percentage of studies with a specific (high, low, and uncertain) risk of the bias according to the (adapted) checklist for Quality Assessment of Diagnostic Accuracy Studies (QUADAS) (33).

To assess the risk of bias in these studies, we adapted the checklist for QUADAS (33). CFT and AP independently evaluated the articles with respect to 12 questions (Figure 3). In the following analysis of our evaluation, we found a 95% agreement.

We identified a high risk of selection bias, as, in the majority (28/30) of studies, the study population was a convenience sample. Study animals were either recruited on specific farms (commercial or research) or in animal hospitals or sanctuaries. If the selection of the study population involves evaluating a diagnostic test, the generalizability of the results may be limited. Sample size calculation was not reported in any study. Control groups were found to be not appropriate in 12 studies. Animals in the control group should be handled in the same way as the treatment group to exclude as many confounding variables as possible. Twenty-two studies used the same animal as control (pretreatment and posttreatment), where the effect of time, habituation, and other variables could influence the facial score (35). We agreed that, in most studies, the intervention produced or relieved pain as the target condition.

The number of observers in the studies were appropriate, as two observers are needed for testing interobserver reliability (36). More than two observers assessed FAUs in the majority of studies. The selection of observers included both genders and different levels of experience and expertise with the species of interest; we rated a low risk of selection bias in this respect. Most authors reported that the observers were blinded to the treatment, but in 16 out of 20 studies using videos or frames, the selection of these was not blinded. Pain assessment with other methods as the reference was performed in 24 studies. If the observer is aware of the result of this additional assessment, this can influence their judging in facial expression scoring (33). This issue was not addressed in these studies.

In most studies, all FAUs scored were included in the analysis. However, some FAUs were not present or very rare in experiments. The authors excluded those from the analysis. Unfortunately, there was no consensus among the studies on the number of FAUs in a composite score or the scale range. As such, a one-to-one comparison of the study results is not possible. The validity, a core criterion for the quality of a diagnostic test, was evaluated in only five studies. Without data on test accuracy, an evaluation of the test quality is not possible.

## Discussion

Animals cannot communicate verbally regarding their perception of pain or distress. To ensure the good welfare of animals under human care, it is essential to be able to recognize and assess pain or distress. This is true for animal husbandry and veterinary issues, especially in animal experiments. For animal experiments, the EU Directive 2010/63/EU requires the assessment of the severity of all procedures in an experiment. In this context, severity describes all adverse effects that animals may experience in an experiment, including discomfort, pain, distress, fear, nutritional deprivation, and behavioral deprivation (37).

As Langford et al. (21) introduced a grimace scale for pain assessment in laboratory mice, a variety of studies aimed at the development and validation of facial expression scores or grimace scales in a variety of species (10) [for a review, see Mota-Rojas (38)]. Although facial expression scores seem to offer an easy-to-learn and cheap pain assessment method, they are not yet widely integrated into the daily routine of animal research (38). These authors concluded that, in their review on grimace scales in laboratory animals, currently, the retrospective character and time-consuming implementation can hinder the establishment of grimace scales in research practice. In our systematic review, we focused on large domestic animals. The housing and handling of large domestic animals are substantially different from those of laboratory animals. This can have an influence on the usefulness and effectivity of a pain scoring system. We wanted to assess the validity and repeatability of this method for large domestic animals and identify the best practice for veterinary practice and farm animals in research.

Following our search criteria, we included 30 articles, the majority of which were published by European working groups. A systematic literature search is always a snapshot of the date of the search (20 January 2022). Therefore, more recent papers are not included in this review.

The EU Directive EU/2010/63 demands valid methods for assessing pain in animals used in experiments. Although farm animals are regularly included in animal experiments, we found only a few articles focusing on large domestic animals as experimental animals. Equids (horses and donkeys) are the major species in the included articles. In the human–animal relationship, speciesism is described. Different motivations to keep an animal have been suggested: instrumental, empathy, or identification; and values or beliefs (39). The attitude of how humans treat an animal depends on the culture of the person, the type of animal, and the function of the animal (40). Companion animals, such as horses, fall into the empathy group, whereas farm animals usually count for the instrumental group. This could lead to a greater interest in a reliable pain assessment tool for horses.

The objective of most studies was to develop a scoring system based on facial expressions. Furthermore, some groups aimed at the validation of these scores as pain assessment methods. For the quality assessment of the studies included in this review, we adapted the Diagnostic Accuracy Studies (QUADAS) checklist (33) to analyze the potential risk of bias. Reliability and validity describe the quality of a test. Interobserver reliability is the consistency of results between different observers, whereas intra-observer reliability refers to the consistency within one observer when evaluating repeatedly. To test the interobserver reliability, at least two observers are needed (36). The number of observers included in the study was stated in 28 studies, and in five studies, only one observer was assessed and no interobserver reliability was tested. Observer variability assessment is calculated by ICC. An ICC ≥ 0.7 is accepted as sufficient (41); this was reported in 5 out of 18 studies investigating interobserver reliability for all FAUs.

Facial expression scoring is promoted as an easy-to-learn test method (42). Previous work experience or qualification of the observer should have no influence on the reliability of the test. Observers with a wide range of experiences are needed for reliability testing to avoid selection bias. In this context, a selection bias would arise if the experience or qualification of the observers would influence their ability to score FAU. The authors found that, in 70% of the studies, the selection of observers was appropriate. Their qualification ranged from no experience to animal professionals, animal scientists, and veterinarians (27). Navarro et al. (43) found no effect on interobserver reliability related to the level of pig experience of the observer. This is in agreement with Mullard et al. (44). They found no influence of professional background in scoring ridden horses. Dai et al. (42) showed that only 30 min of training significantly improved the agreement between the observers; training observers had a great variability in scoring horse FAUs. Additionally, Navarro et al. (43) reported an effect of the gender of the six observers on the score, with the four female observers having higher reliability than male observers. For any best practice guideline, before using facial expression scoring, observers should receive specific training for scoring systems to ensure reliable results (42).

Facial expressions of pain in humans are characterized by lowering of the eyebrows, squeezing of the eyes, wrinkling of the nose, raising of the upper lip, and opening of the mouth (45). Equivalent FAUs were implemented for assessing pain in large domestic animals [as in laboratory animals (21)]. The assessment included FAUs in the eye area, the orbital region, the cheek or the chewing muscles, the lips, the mouth, and, in addition to the human pain face, the position of ears.

The ability and reliability to score a respective FAU varied considerably (Figure 2). In horses, the evaluation of “ear position” seemed easy, but 21% of observers noted “not able to score” for tension above the eye, strained mouth, and pronounced chin (26, 46). The frequency of appearance of the FAUs also had an influence. When only moderately presented, Czycholl et al. (47) could not detect any reliability for “orbital tightening” or “tension above the eye area” in a study on welfare assessment in horses. In pigs, “orbital tightening” was easy to recognize for the observer, whereas 72% had difficulties with “nostril dilatation” (27). In sheep suffering painful clinical diseases, all five FAUs investigated seemed to be easy to score, with a maximum of 12% “not able to score” for orbital tightening (25). However, the agreement was low in lambs undergoing tail docking, when scoring “mouth changes” and “cheek flattening” in contrast to the strong agreement for “ear posture” (48). This can imply that age and the type of painful condition can influence the visibility of specific FAU. The agreement for FAU “ear position” was a “very good” agreement [ICC 0.81–1, (34)], whereas the agreement for all other FAUs varied between moderate (0.41–0.6) and good (0.61–8.0, Figure 2).

It seems that the ear position is easy to score, whereas tension above the eye, orbital tightening, and FAU around the mouth are sometimes difficult to score, which can affect the reliability of these specific FAUs. Giving the scores with higher reliability, more power in a composite score might improve the overall reliability.

Twenty-one studies in this review scored pictures captured from videos of the study animals. This procedure has limitations. First, there is a substantial risk of bias as only 20% of the authors reported that the selection of the frames was blinded or done by a person not familiar with FAU scoring. There is a risk that frames are selected with respect to the prominence of specific FAUs. Next, these pictures represent the face of an animal only for a fraction of a second. This bears the danger of missing important FAU activities. Gleerup et al. (49) remarked that facial expressions were altered during pain induction and that not all features identified were present simultaneously at all times. As such, a frame would express a different pain face rather than a live image over time. Dalla Costa et al. (50) found no significant differences in the horse grimace scale between still images and 15-s video sequences, but they had a higher variation of scores between the observers when scoring videos. In laboratory animals, live grimace scores were found to be significantly lower than retrospective scores of still images or videos (51, 52). This is in agreement with the findings of Conegelian et al. (53) evaluating dental pain in horses. In their study, pain scores evaluated in motion were always lower than scores from photographic evaluators. Thus, it seems that facial expression scoring in pictures has different challenges from scoring in real time or videos, and each method potentially has to be assessed separately. To establish facial expression scoring as a pen-side pain assessment, validation has to be done under field conditions as well. Further research is required here. A pain assessment method is only valuable for clinical decision-making, when the result is promptly available while examining the animal rather than retrospectively. In laboratory mice, some research has been done to automate frame selection (54) to enhance the effectivity of pain scoring, research on the use of the algorithm for facial expression scoring in farm animals is only limited (55, 56).

Another limitation of pictures or videos for scoring is the selection process. Although the observers were blinded to the treatment, the selection of pictures or videos was sometimes not blinded. These studies are at risk of overestimating the presence of FAU characteristics for pain. This can also happen if persons with expertise select photographs or videos for evaluation in facial expression scoring.

A test method not only has to be reliable but also valid. Validity is a measure of how accurately a test system describes the real situation (57). Testing “true” or criterion-related validity needs a gold standard as the reference. Pain is a subjective experience, and animals cannot express themselves verbally. Approximately 75% of the studies in this review had difficulties in defining a reference gold standard method, so there is a substantial risk of verification bias. In the absence of an error-free reference standard, a gold standard construct or content validity is a possible measure to describe the quality of a pain test for animals. These methods compare the results of the test to be evaluated with other indirect test methods (i.e., cortisol measurements or behavior assessment) or with specific plausible procedures (i.e., castration or tail docking), respectively. All studies in this review that tested validity compared the results of scoring FAU to painful diseases or surgical intervention as the reference of pain.

There are challenges to pain scoring systems as pain has multiple dimensions. Two of these dimensions are intensity and length. Van Loon et al. evaluated a chronic pain score for horses and donkeys (58, 59). While the chronic pain scale identified pain in chronically diseased donkeys, it was not so reliable for horses with chronic pain. Also, in humans, facial expressions of chronic pain are challenging. The lack of a pain-free baseline for comparison and the overload of emotional components make it difficult to describe a chronic pain face (15).

Methods of assessing pain intensity are needed for adequate pain management. Human subjects were asked to describe their pain experience on a scale from 1 to 10 (4). Based on the studies in this review, animals were classified as either in pain or pain-free. The framework of Directive 2010/63/EU demands a classification of the animal's burden in the experiment into low, middle, or severe. Further research is warranted if pain intensity can be evaluated with facial expression scoring.

## Conclusion

Facial expression scores or grimace scales have been developed for a wide range of species, including large domestic animals. This review revealed that the reliability of these scores is satisfactory. In the majority of the studies, it was demonstrated that facial expressions changed during painful events. To ensure substantial reliability, observers should receive training on the scoring system. Composite scores should consider that some FAUs are easier to score and occur more frequently than others. The assessment of the validity of grimace scales continues to be challenging. Before implementing facial expression scoring as a real-time assessment method, further validation of live scoring is still needed. Overall, the facial expression score seems to be suitable for identifying animals in acute pain even though the validity of measuring the intensity of pain has not been validated yet.

## Data availability statement

The raw data supporting the conclusions of this article will be made available by the authors, without undue reservation.

## Author contributions

Conceptualization and writing—original draft preparation: CF-T. Validation and writing—review and editing: CF-T, JM, and AP. Formal analysis: CF-T and AP. Data curation: AP. All authors contributed to the article and approved the submitted version.

## Conflict of interest

The authors declare that the research was conducted in the absence of any commercial or financial relationships that could be construed as a potential conflict of interest.

## Publisher's note

All claims expressed in this article are solely those of the authors and do not necessarily represent those of their affiliated organizations, or those of the publisher, the editors and the reviewers. Any product that may be evaluated in this article, or claim that may be made by its manufacturer, is not guaranteed or endorsed by the publisher.
